# Dr. Daniel D. Neff

**Published:** 1914-12

**Authors:** 


**Dr. Daniel D. Neff,** Bellevue 1894, died of poison, self-administered it is believed, at his home in Syracuse, October 13, aged 45.

